# Interleukin-23 promotes the epithelial-mesenchymal transition of oesophageal carcinoma cells via the Wnt/β-catenin pathway

**DOI:** 10.1038/srep08604

**Published:** 2015-02-27

**Authors:** Deyu Chen, Wei Li, Shenzha Liu, Yuting Su, Guohu Han, Chenchen Xu, Hongli Liu, Tingting Zheng, Yuepeng Zhou, Chaoming Mao

**Affiliations:** 1 Institute of Oncology, the Hospital Affiliated to Jiangsu University, Zhenjiang. 212001, China; 2 Department of Nuclear Medicine, the Hospital Affiliated to Jiangsu University, Zhenjiang. 212001, China; 3 Department of Oncology, Jintan Hospital Affiliated to Jiangsu University, Changzhou. 213200, China

## Abstract

As the eighth most common malignant tumour worldwide, oesophageal cancer (OC) is often diagnosed during the metastasis of its advanced stage. Interleukin (IL)-23 is an immunomodulatory cytokine that has recently been identified as a cancer-associated factor. However, the role of IL-23 in the evolution of OC remains unclear. In the present study, we found that IL-23 was significantly expressed in the tumours of OC patients suffering metastasis and demonstrated that IL-23 contributed to epithelial-mesenchymal transition (EMT) through the Wnt/β-catenin pathway, promoting the migration and invasion of OC cells. In conclusion, IL-23 plays a pivotal role in the development of OC via EMT.

In 2008, approximately half a million cases of oesophageal carcinoma (OC) were diagnosed worldwide, and more than 400, 000 people died of OC^1^. In China, mainly oesophageal squamous carcinoma, was the 4th leading cause of cancer deaths during 2009, consistent with the substantial morbidity and the shocking number of sufferers^2^. Due to the continual improvement of therapy technologies and successful clinical applications, locally aggressive tumours at the early stage can be cured. However, in reality, OC is often diagnosed at an advanced stage with distant metastasis because that the early symptoms vary in intensity and the progress rapidly, making the 5-year survival rate only 35–45% for all patients with OC^3^,^4^. Undoubtedly, the invasion and metastasis of tumour cells are complex and intricate, and the epithelial-to-mesenchymal transition (EMT) is an important milestone during the development of the epithelial cancers^5^. After the transformation in phenotype and the acquisition of functions, the cells begin to attack the extracellular matrix and vasculature, move to distal tissues and organs, recruit support and induce angiogenesis and lymphangiogenesis, thereby forming metastases. Moreover, EMT induces changes in the expression of epithelial and mesenchymal genes such as snail and slug or negative factors such as miRNA200s, including decreases in the epithelial marker E-cadherin and increases in the mesenchymal marker vimentin^6^,^7^,^8^,^9^,^10^. Notably, tumour cells usually undergo this malignant transformation with concomitant changes in MMPs (matrix metalloproteinases), VEGFs (vascular endothelial growth factors) or other similar proteins^11^,^12^. Because cancer cells acquire malignant behaviour through the complex interplay of a number of factors and specific microenvironments, targeting molecules associated with the EMT program is of significant therapeutic interest for resisting invasion and metastasis.

As an indispensable part of the tumour microenvironment, immune cells and their roles are gaining growing attention^13^,^14^. According to recent studies, tumour heterogeneity refers to not only cancer cells of different functions and characteristics but also to stromal cells and helper cells, sometimes including immune cells. Our previous studies suggested that once Th17 cells (T helper cell 17) infiltrate oesophageal squamous cell carcinoma, differentiation-related cytokines (IL-23, IL-1β, and IL-6) can be detected in the microenvironment^15^. The process of tumour development includes changes in the populations of immune cells and in the expression of associated factors. For example, a major product of macrophages and dendritic cells, the integrated IL-23 (IL-23p19 and IL-12p40), plays a crucial role in the late developmental functions of Th17 cells^16^. Recent studies on IL-23 focus on the risk of autoimmune inflammation diseases, including rheumatoid arthritis (RA) and psoriasis, considerably improving not only the understanding of molecular pathogenesis but also the development of potential therapeutic targets^17^,^18^,^19^. However, the role of IL-23 in cancer is not consistent and is occasionally controversial. In some studies, IL-23 displays anti-tumour and anti-metastatic effects, whereas in others, it promotes proliferation and metastasis^20^,^21^,^22^,^23^,^24^,^25^,^26^,^27^,^28^. Therefore, the elucidation of how IL-23 impacts cellular homeostasis is urgent. Members of the Wnt/β-catenin pathway are secreted molecules that influence cancer development by regulating EMT and metastasis. Mutations in glycogen synthase kinase-3β (GSK3β) and in β-catenin, the key mediators of Wnt/β-catenin signalling, are found in a majority of cancers, underlying the dysregulation from the influence of the microenvironment^29^,^30^,^31^,^32^. When the Wnt pathway is activated, cytoplasmic β-catenin is no longer degraded by GSK3β, and large amount of β-catenin translocates to the nucleus, where it interacts with lymphoid enhancer/T-cell transcription factors (TCF/LEF) and cAMP-response element binding protein-binding protein (CBP), thereby regulating the transcription of genes such as E-cadherin, MMP or VEGF^33^,^34^,^35^.

Much evidence shows that IL-23 can promote proliferation and metastasis in lung, colorectal and oral cancer^24^,^25^,^26^,^27^,^28^, however, it remains controversial whether IL-23 contributes to EMT. Clinical diagnoses show that, OC is quite prone to metastasis and formation of new vessels, but the relevant studies are scarce. However, there is no recent data available connecting IL-23 and the EMT and tumour metastasis in OC. Furthermore, the signalling pathways involved in this process have not yet been investigated. Thus, in the present study, we investigated the effects of IL-23 regulation on EMT and the underlying signalling pathways in oesophageal carcinoma.

## Results

### The distribution of IL-23 in tumour tissues is correlated with metastasis

**Figure 1:**
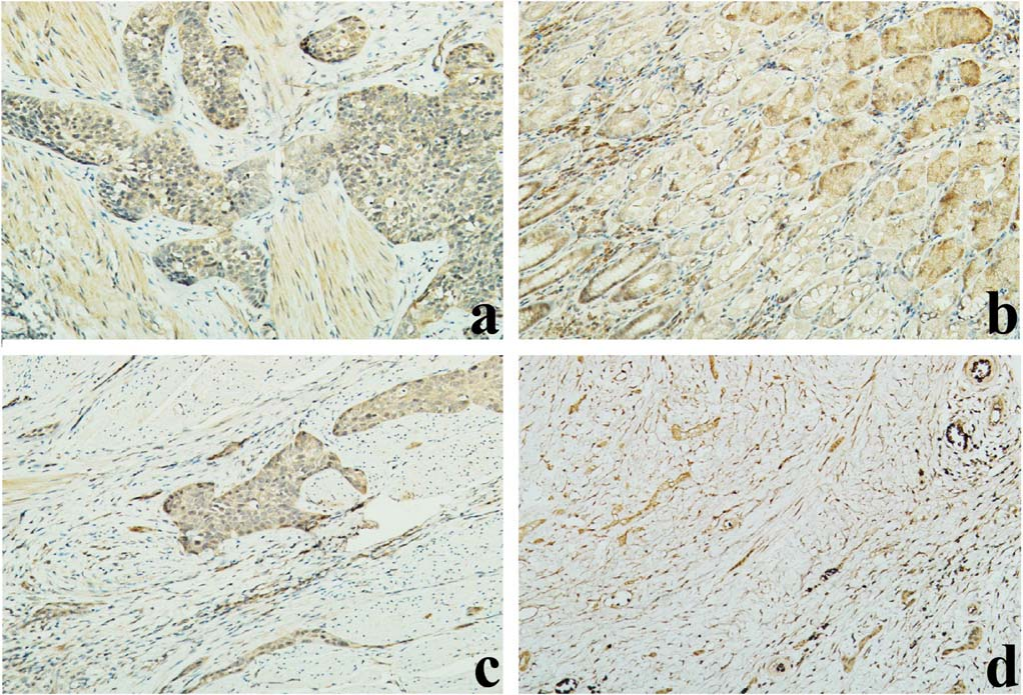
Distribution of IL-23 in patients with OC. Representative images of IL-23 expression in metastatic tumour (a) and precancerous tissue with metastasis (b) by IHC staining (200 × magnification). Representative images of IL-23 expression in primary tumour (c) and precancerous tissue without metastasis (d) by IHC staining (200 × magnification).

Comparing the area of IL-23 expression in tumours with and without metastasis and in precancerous tissues, we found a connection between the secretion intensity and the malignancy; that is, IL-23 was expressed in both tumour and precancerous tissues, and the IL-23R was distributed on the cells membranes (Figure 1a, b, c, d and S1). In 10 OC cases without metastasis, no significant difference (*p* = 0.087) was observed in the size of the IL-23-positive area between tumours and precancerous tissues. However, in 13 OC cases with metastasis, the IL-23-positive area was significantly larger (*p* = 0.025) in tumour tissues than in precancerous tissues, and metastatic tumour tissues showed significantly larger IL-23-positive area than primary tumour tissues (*p* = 0.043). Further, IL-23-positive area was significantly larger in precancerous tissues from metastatic than non-metastatic cases (*p* = 0.031) (Figure 1a, b, c and d).

Using the merged scores from two experienced pathologists who were blinded to the conditions, the intensity of IL-23 staining was analysed according to the specific judgement standard as described (in the immunohistochemical staining of the Methods). The expression intensity of IL-23 was independent of patients' age, gender, tumour size, tumour location and TNM stage, whereas the area of IL-23 expression was significantly correlated to patients' lymphatic and metastatic status (Table 1).

### IL-23 is involved in the regulation of EMT

**Figure 2:**
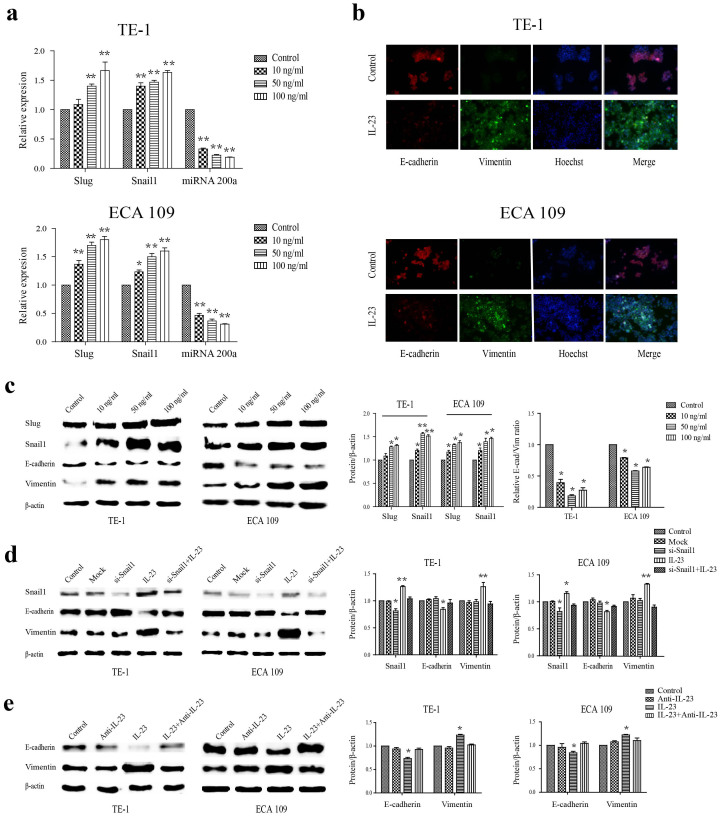
Expression of the EMT in OC cells cultured with IL-23. (a) Relative expression levels of slug, snail1 and miRNA 200a after the treatment with different concentrations of IL-23 (0, 10, 50, 100 ng/ml) by RT-PCR. The analysis of the CT values of oesophageal cancer cells was normalised to GAPDH for each sample. The normalised values (ΔCT) of all samples were compared with the control in each group (ΔΔCT). The results are expressed as 2-ΔΔCT, and the resulting data derive from at least three independent experiments. (b) Immunofluorescence: Cells were treated with 50 ng/ml IL-23 for 24 h and then stained for E-cadherin and vimentin. E-cadherin: red, vimentin: green, cell nuclei: blue. (c) Western blotting for slug, snail1, E-cadherin and vimentin: After the treatment with various concentrations of IL-23 (0, 10, 50, 100 ng/ml) for 24 h, the cells were lysed for western blot analysis. Quantification of the western blot results showing the changes in snail1 and slug expression and the E-cadherin/vimentin ratio. E-cad/Vim: E-cadherin/Vimentin. (d) and (e) The changes in E-cadherin and vimentin expression after pretreatment with siRNA targeting snail1 or in the level of IL-23 (50 ng/ml) after treatment with anti-IL-23 (1 μg/ml) by western blotting. Quantitative analysis of panels is showed in the bar chart. All data derive from at least three independent experiments. Mock: transfection with lipofectamine 2000 without siRNA, si-Snail1: siRNA snail1. *p < 0.05, **p < 0.01.

In this study, we assessed the progress of EMT using the promoters slug and snail1, and the miRNA 200a as a negative marker. We used RT-PCR to examine the expression of those mRNAs in TE-1 and ECA 109 cells in the presence of IL-23. Both slug and snail1 were markedly up-regulated, whereas miRNA 200a was sharply down-regulated, in both TE-1 and ECA 109 cells (Figure 2a). The immunofluorescence assay in Figure 2b shows that after the IL-23 treatment, the markers of the transition were detected and confirmed stable. After treatments with varying amounts of IL-23 (0, 10, 50, 100 ng/ml), western blot results showed a decrease in the level of E-cadherin along with distinct increases in the levels of vimentin, slug and snail1 expression in TE-1 or ECA 109 cells in a dose-dependent manner, and the ratio of E-cadherin/vimentin was significantly decreased (*p* < 0.05) (Figure 2c). However, to directly confirm that the regulation of IL-23 depended on the above promoters, siRNA-based gene silencing was used. Snail1 knockdown was confirmed by quantitative PCR. Specifically, after 12 h, 24 h, 48 h and 72 h, TE-1 and ECA 109 cells exhibited 39.2 ± 1.6%, 71.1 ± 1.3%, 55.9 ± 2.1% and 32.3 ± 3.4% reduction (Supplementary Figure S1). Without the existence of snail 1, even with the same concentration of IL-23, the occurrence of mesenchymal transition had been reduced but not completely abrogated (Figure 2d). To further clarify the role of IL-23, a neutralising antibody against IL-23 was tested. With the neutralisation of IL-23, cells maintained same ratios of E-cadherin/vimentin as controls (Figure 2e). These findings confirm that the IL-23 caused the transformation from the epithelium to the mesenchyme in OSCCs.

### IL-23 induces the activation of the Wnt/β-catenin pathway to generate metastasis

**Figure 3:**
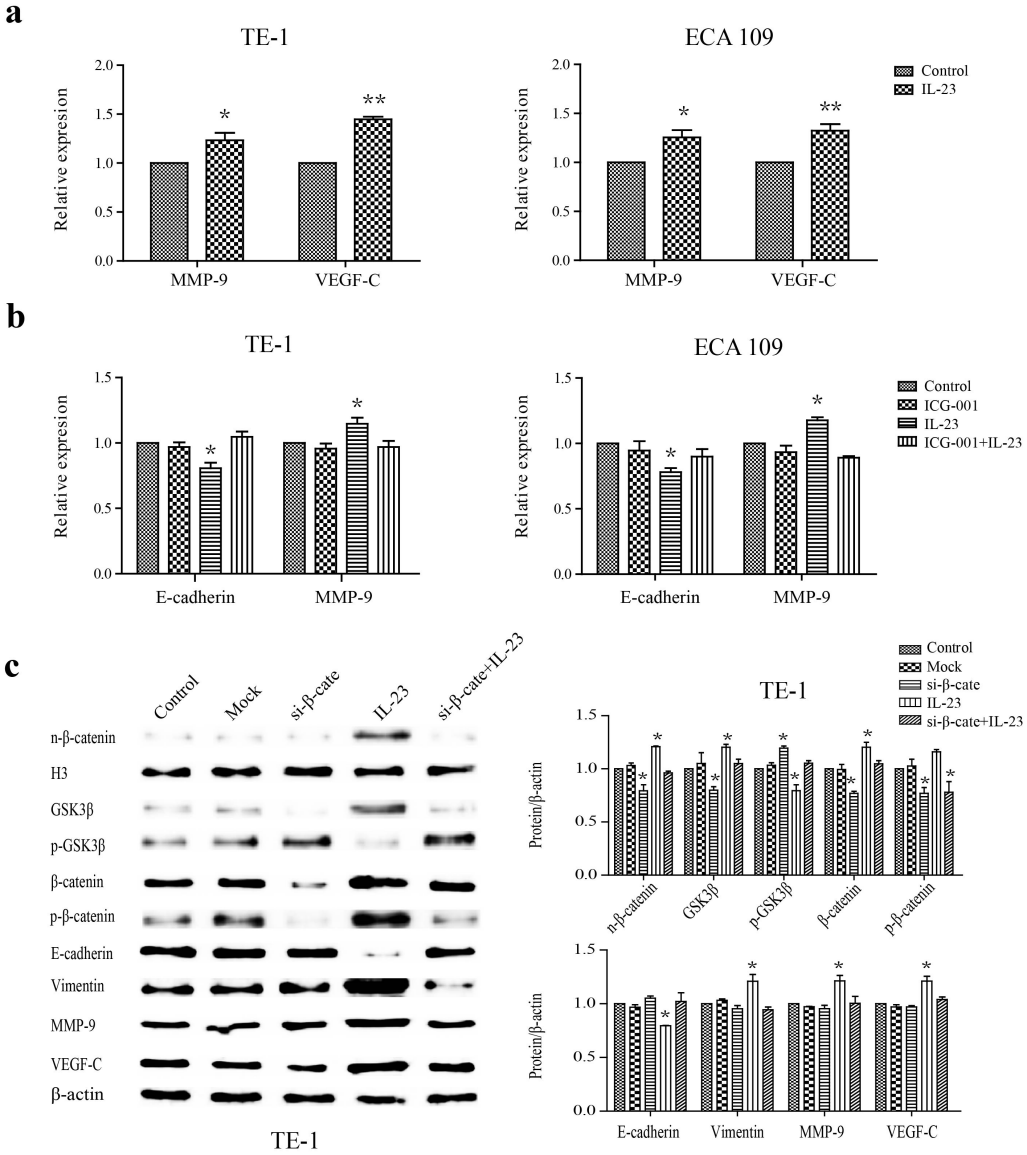
The expressions levels of Wnt/β-catenin and metastasis related-proteins in OSCCs treated with IL-23. (a) Effect of IL-23 on MMP-9 and VEGF-C expression: TE-1 and ECA 109 cells were treated with IL-23 (50 ng/ml) for 24 h. The supernatants were then collected for ELISA analysis. (b) The mRNA levels of E-cadherin and MMP-9 after treatment with ICG-001 (5 μM) and/or IL-23 (50 ng/ml); (c) Western blotting results showing the changes in n-β-catenin, GSK3β, p-GSK3β, β-catenin, p-β-catenin, E-cadherin, vimentin, MMP-9 and VEGF-C expression levels in TE-1 cells treated with IL-23 (50 ng/ml) for 24 h andβ-catenin silencing. Histone H3 andβ-actin were used as protein loading controls. Statistical results are presented as the means ± SEM (n = 3) based on ANOVA followed by the Tukey-Kramer test. Quantitative analysis of panels is showed in the bar chart. Experiments were repeated at least three times with similar results, and only one representative result is shown. Mock: transfection with lipofectamine 2000 without siRNA, si-β-cate: siRNA β-catenin, n-β-catenin: nuclear β-catenin, p-GSK3β: phospho-GSK3β, p-β-catenin: phospho-β-catenin, H3: Histone H3. *p < 0.05, **p < 0.01.

Because EMT is accompanied by stromatolysis, invasion and metastasis, we next investigated the effect of IL-23 on the expression levels of MMP-9 and VEGF-C in OC cell lines. The secreted levels of MMP-9 or VEGF-C expression were upregulated in TE-1 or ECA 109 cells treated with IL-23 compared to control, suggesting that the IL-23 might contribute to tumour metastasis (Figure 3a). Wnt/β-catenin has been reported as a key signalling pathway of malignant biological behaviours in many cancers, and it can regulate the expression levels of E-cadherin, MMP-9 and VEGF-C. We therefore tested whether the effects of IL-23 on these proteins in OC cells were also mediated by the Wnt/β-catenin pathway. The addition of ICG-001 (5 μM), a Wnt/β-catenin inhibitor targeting the CBP, to the medium before IL-23 treatment, the decrease of E-cadherin and the increase of MMP-9 as the activated symbol of Wnt were significantly blocked at the mRNA levels^35^ (Figure 3b). The siRNA-mediated downregulation of β-catenin, which not only combines with E-cadherin to play a direct role in adhesion but also acts as the pivotal node in the Wnt pathway, demonstrates that the Wnt/β-catenin pathway acts directly on IL-23 induced EMT and the subsequent metastasis. The level of the nuclear β-catenin was dramatically elevated in TE-1 and ECA 109 cells after IL-23 treatment (50 ng/ml), along with an increase in p-GSK3β, but there was no apparent change in the level of p-β-catenin and the stimulative of vimentin, MMP-9 and VEGF-C. However, the absence of β-catenin would block the effect of IL-23, and the density of p-GSK3β, p-β-catenin and nuclear β-catenin showed no greater change than observed in cells which β-catenin alone was inhibited (Figure 3c; similar results from ECA 109 cells not shown). The β-catenin knockdown cells showed the effective transfection at mRNA levels by 81.3 ± 1.9% at 24 h (Supplementary Figure S1). In conclusion, IL-23 inactivated p-GSK3β, further decreased the phosphorylation of β-catenin inducing its enrichment in the nucleus, and caused the activation of target genes that induce the EMT and metastasis.

### IL-23 enhanced OC cell motility in vitro and vivo

**Figure 4:**
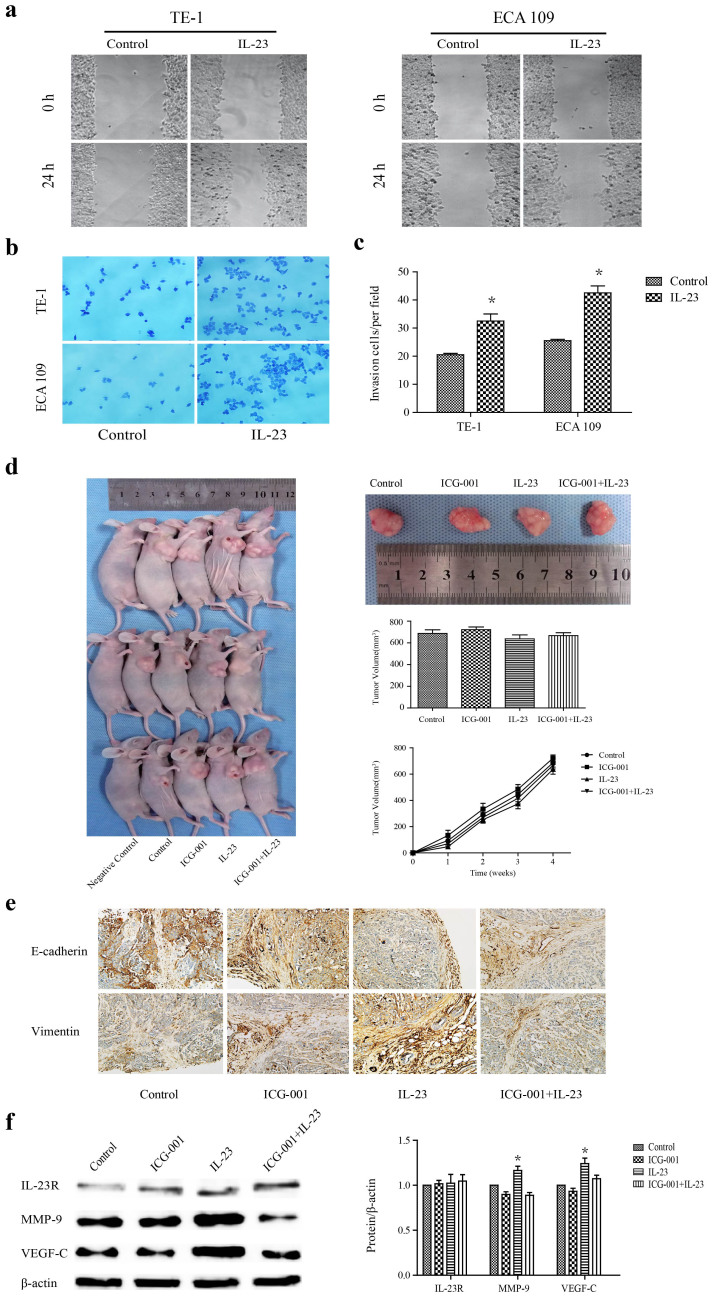
IL-23 enhanced OC cells motility. Confluent monolayers of TE-1 and ECA 109 cells were wounded and the migratory capacity of the cells was measured at 24 h or 48 h by counting the number of cells per field in at least 8 fields from three different experiments. (a) Representative wounds in monolayers of OC cells treated with IL-23 (50 ng/ml) are shown in comparison to untreated controls. (b) The migration-inducing effect of the application of IL-23 in TE-1 and ECA 109 cells. (c) Quantification of the invasion-inducing effect of IL-23-treatment (50 ng/ml). (d) The results showing the changes in volume and terminal states of tumours. (e) Immunohistochemical results show the transformation due to IL-23 in xenograft tumours, using E-cadherin and vimentin as the indicators. (f) The collected tissue homogenates were used to detect the levels of IL-23R, MMP-9 and VEGF-C by immunoblotting. Data represent mean values from at least three independent experiments. Asterisks indicate the level of significance in the charts. *p < 0.05.

The effect of IL-23 on cell motility was first investigated through wound-healing and Matrigel invasion assays in vitro. The wound-healing assay and transwell migration assay showed that, compared to control parental cells, IL-23 (50 ng/ml) could markedly increase the cell migration rate at the edge of the scratch in both TE-1 and ECA 109 cells (Figure 4a and b). Moreover, the invasion assay showed that the invasiveness of cells treated with IL-23 (50 ng/ml) was significantly higher than that of control parental cells (*p* < 0.05) in all tested cell lines (Figure 4c).

To assess the role of IL-23 in the elicitation of EMT and invasion, we introduced TE-1 tumours into BALB/c nude mice, with or without selective ablation of the activation of Wnt by ICG-001. Figure 4d shows that the growth rates and volumes of xenograft tumours were similar in both lines of mice, and the largest tumour was detected after treatment with ICG-001 alone. Consistent with a critical role for Wnt in producing EMT, immunohistochemical assays showed that IL-23 disrupted Wnt signalling and promoted tumour infiltration and invasion through vessels, which had been shut down by the ICG-001 pretreatment (Figure 4e). With the finding that tumour cells express functional IL-23R, MMP-9 and VEGF-C after the treatment with IL-23 and/or ICG-001, we found that IL-23 can promote tumour metastasis by the activation of Wnt. In mice bearing tumours, the injection of ICG-001 alone did not change the cancer cells' behaviour, but pretreatment with ICG-001 reduced the increase induced by IL-23. Further confirming the in vitro results, the levels of IL-23R were steady in mice, regardless of the treatment group (Figure 4f). These data demonstrate that IL-23 enhanced the migration and invasive ability in OC.

## Discussion

Recent investigations have demonstrated that IL-23 plays a role in tumorigenesis and acts as a potential marker of the invasion process^24^,^25^,^26^,^27^,^28^. Here, we found that IL-23 was highly expressed in samples from OC patients, especially with the progression of malignancy. As an immunomodulatory factor, the heterodimeric cytokine IL-23 is mainly secreted from mature macrophages and dendritic cells, which indicates that the effects of immune cells and their factors in tumour action are varied. The proliferation and metastasis of cancer cells give rise to the growth and propagation in tumour, and IL-23 may participate in that process by regulating of the expression and modification of STAT3/5 and NF-κB (p65, RelA), with different effects in different types of cells^25^,^26^,^27^,^28^. However, the associations between IL-23 and EMT susceptibility have not been assessed in any OC.

In this work, we quantitatively tested the regulating transcription factors during EMT in OSCCs induced by IL-23. The increasing expression of slug and snail1 showed a strong correlation with the loss of miRNA 200a after treatment, suggesting that IL-23 is a likely agonist of EMT. Snail1 and slug, when overexpressed in epithelial cells, have been described as direct repressors of E-cadherin and inducers of EMT or invasion^6^, and they are also associated with prognosis in oesophageal cancer. MicroRNA 200a, a member of the miRNA-200 family, plays a major role in inhibiting the epithelial mesenchymal transition in EMT-like phenotypes and shows the opposite correlation with OC prognosis. This miRNA targets transcriptional repressors of E-cadherin in various cancer cells, such as gastric adenocarcinoma, glioma, breast cancer, and lung cancer^9^,^10^. Furthermore, whereas the expression levels of E-cadherin decrease, the levels of vimentin, slug and snail1 increase markedly, causing further dysfunction in cells adhesion and adherens junctions, thereby allowing the dissemination of tumour cells. We therefore measured the ratio of E-cadherin/vimentin after silencing snail1 or neutralising IL-23. IL-23 can promote the occurrence of EMT in OC cells. After the cancer cells lost epithelial characteristics, they acquired the invasive ability to pass through membranes. To further study the progress and extent of the epithelial-mesenchymal transition, we used the in vitro migration and invasion assay, investigating the expression of epithelial and mesenchymal markers along with proteins indicating metastasis in xenograft tumours. Based on these significant and validated results, we demonstrate that IL-23 is both a marker for metastatic tumours, and also directly promotes the invasion and migration of tumour cells. This implies that IL-23 is a potential cause as well as a functional biomarker for EMT and metastasis in OC.

To assess how IL-23 drives the OC cells to invade through basilar membranes and metastases, we hypothesised that the Wnt/β-catenin signaling pathways would be activated by IL-23. Once the extracellular matrix, the first line of defence against the metastasis of tumour cells, was breached, cancer cells can metastasise through vessels. The current results show that, as an adhesion molecules, β-catenin plays a key role in the interaction between cells and the extracellular matrix, and abnormally high expression of β-catenin has been associated with malignant behaviour in multiple cancers^30^,^31^,^32^,^35^. The current data show that knockdown of β-catenin expression in cancer cells decreased the expression of snail1, vimentin, MMP-2, MMP-9, VEGF-A and VEGF-C, indicating that the Wnt/β-catenin signaling pathway regulated EMT and metastasis. Through the degradation of the extracellular matrix and vascular basilemma, as well as the active regulation of VEGF, MMPs promote and contribute to tumour infiltration and metastasis^29^,^30^,^31^. By silencing the expression of β-catenin, we found that IL-23 supported the function of β-catenin via the modification of GSK3β, followed by increases in MMP-9 and VEGF-C, which further directly increased metastasis in vitro. To further confirm the role of Wnt in vivo, we tested the molecular inhibitor ICG-001^35^. The exogenous IL-23 had transformed into a strong environmental factor stimulating EMT and invasion, and its effects could not be separated from the induction by activated Wnt. Thus, our results confirm that IL-23 regulates the cell infiltration and invasion via the Wnt/β-catenin pathway.

Previous studies of IL-23 focused on the risk of autoimmune inflammation, and demonstrated that the cellular function of IL-23 was associated with the self-reactive products, playing a critical role in the development of autoimmune inflammation^17^,^18^,^19^. However, the role of IL-23 in tumours has remained confusing^20^,^21^,^22^,^23^,^24^,^25^,^26^,^27^,^28^. This is likely because tumours are not homogeneous, including not only tumour cells of unequal differentiation and function but also other stromal cells and immune cells, and there is further confusion between the IL-23p19 secreted by cancer cells and the normal function of IL-23. The tumour heterogeneity constitutes the microenvironment, which affects the tumourigenicity, the development and the prognosis of the tumour; therefore, the effects of immune cells and relevant environmental factors are receiving serious attention. Our results show that IL-23 itself was a high-risk index for OC. The other results regarding associations between the IL-23R susceptibility and ESCC were obtained from a case-control study derived from an eastern Han Chinese population^36^. Based on the supplementary data, we also demonstrate that IL-23 could neither stimulate proliferation through IL-23R, as reported by other studies, nor raise the endogenous expression level of IL-23p19 in OC cells (Supplementary Figure S2, S3, S4), proving that the test data were proper and reliable. Our results suggest that IL-23 can stimulate the epithelial-mesenchymal transition and further metastasis of the oesophageal squamous cell carcinoma and that this process likely occurs via Wnt/β-catenin pathway.

## Methods

### Patient samples

Oesophageal cancer and the adjacent non-cancerous tissues were collected from 23 patients at the Hospital Affiliated to Jiangsu University (China). The tumours of patients had undergone neither chemo-radiotherapy nor surgery, which was confirmed at the Department of Pathology in the Hospital Affiliated to Jiangsu University. The precancerous tissues were obtained from tissues 5 cm away from the tumour margin, as reported previously^37^. All tissues were collected and immediately stored at −80°C before further analysis. All patients provided written informed consent in accordance with the guidelines of the Ethics Committee of The Affiliated Hospital of Jiangsu University.

### Immunohistochemical (IHC) staining

Immunohistochemical staining with antibodies against IL-23, E-cadherin and vimentin (1:100 dilutions) was performed as follows: The samples were fixed in 10% paraformaldehyde, embedded in paraffin, sectioned into 4 μm sections, and mounted on slides. After being deparaffinised, rehydrated, antigen unmasked and immersed, the slides were washed with PBS, blocked with 10% normal goat serum, and then incubated with the antibody overnight at 4°C. After three washes, the sections were treated with the corresponding streptavidin peroxidase–conjugated secondary antibody. Tissue sections were then counterstained with 3, 3′-diaminobenzidine (DAB) and haematoxylin and then observed under light microscope. The intensity of IL-23 was recorded at 200 × magnification with an optical microscope, and the differences were divided based on the following evaluation criteria: 3, the area of the matrix was entirely dark brown or was more than 75% brown; 2, the matrix was more than 50% brown; 1, more than 20% of the matrix was brown or light brown. Five fields were randomly chosen from each sample for assessment. All statistics are based on data collected by independent pathologists blinded to the experimental conditions.

### RNA extraction and quantitative reverse transcription–polymerase chain reaction

Total RNA was extracted from TE-1 or ECA 109 cells with Trizol reagent (Invitrogen, USA) according to the manufacturer's instructions. RNA was eluted with RNase-free water and stored at −80°C. RNA concentrations were determined by NanoDrop spectrophotometry. Using random hexamers and oligo dT as a primer, 1 μg of total RNA was reverse transcribed in a 20 μl volume using PrimeScript® RT reagent Kit Perfect Real Time. The qRT-PCR was performed with SYBR® Premix Ex Taq TM (Tli RNaseH Plus) in the Real-time PCR Mx3000PTM System (Genetimes Technology, China). For mRNA detection, the primers were as follows: slug (forward: 5′-AACAGAGCATTTGCAGACAGGTC-3′; reverse: 5′-GCTACACAGCAGCCAGATTCC-3′); snail1 (forward: 5′-CTTCTCCTCTACTTCAGTCTCTTCC-3′; reverse: 5′-TGAGGTATTCCTTGTTGCAGTATTT-3′); E-cadherin (forward: 5′-CAATGCCGCCATCGCTTAC-3′; reverse: 5′-ATGACTCCTGTGTTCCTGTTAATG-3′); MMP-9 (forward: 5′-GGGACGCAGACATCGTCATC-3′; reverse: 5′-TCGTCATCGTCGAAATGGGC-3′); GAPDH(forward: 5′-TCAACGGATTTGGTCGTATTG-3′;reverse: 5′-TGGGTGGAATCATATTGGAAC-3′). GAPDH was used as the internal control. MiRNA-200a was reverse-transcribed to cDNA using the miRNA-specific stem-loop reverse-transcription primer (GENEray, Shanghai, China). The amount of target gene expression (2-ΔΔCt) was normalised via the endogenous small nuclear RNA U6 by using miRNA-specific primers (GENEray, Shanghai, China). The reaction condition were established according to the instructions from GENEray. The qRT-PCR primers were as follows: miR-200a (forward: 5′-ACACTCCAGCTGGGCATCTTACCGGACAGTG-3′; reverse: 5′-CTCAACTGGTGTCGTGGAGTCGGCAATTCAGTTGAGTCCAGC-3′; reverse TRP: 5′-TGGTGTCGTGGAGTCG-3′); U6 (forward: 5′-CTCGCTTCGGCAGCACA-3′; reverse: 5′-AACGCTTCACGAATTTGCGT-3′). The 2-ΔΔCt method was used to calculate the relative levels of gene expression.

### Immunofluorescence

Oesophageal cancer cells, from the TE-1 and ECA 109 lines, were cultivated in 24-well plates and treated with or without IL-23. First, the plates were washed with PBS, fixed in 4% paraformaldehyde at room temperature for 20 min, permeabilised and blocked with 0.1% Triton-X-100 and 5% BSA in PBS at room temperature for 30 min. Next, the cells incubated with primary antibody (E-cadherin or vimentin) overnight at 4°C. They were then incubated with a secondary Cy3-labelled or FITC-labelled antibody at room temperature for 1 h. These stained cancer cells were observed with a fluorescent microscope (Axio Observer, Germany).

### ELISA

To assess the total concentrations of free MMP-9 and VEGF-C after IL-23 treatment, 96-well enzyme immunoassay ELISA kits (eBioscience, San Diego, USA) were used. At 24 h post-incubation, supernatants of the cells were harvested for analysis of cytokine secretions using the ELISA assay kit. Sample preparation, dilution and enzyme detection were performed according to the manufacturer's instructions.

### Transfection with siRNA

Knockdown of snail1 or β-catenin was performed by transfection with small interfering RNA (GenePharma, Shanghai, China). The targeted β-catenin sequences were as follows: sense, 5′-GUCCUGUAUGAGUGGGAACTTdTdT-3′; antisense, 5′-GUUCCCACUCAUACAGGACTTdTdT-3′. The targeted Snail1 sequences were as follows: sense, 5′-CAAACCCACUCGGAUGUGAAGAGAUTTdTdT-3′; antisense, 5′-AUCUCUUCACAUCCGAGUGGGUUUGTTdTdT-3′. A total of 5 × 10^5^ cells were plated in 6-well plates for 18 h and then transfected with 100 pmol of siRNA using Lipofectamine 2000 (Invitrogen, USA) in serum-free medium for 5 h. Then, the medium was replaced with serum-supplemented medium which for 24 h. Negative controls using non-transfected cells and empty-vector were performed in parallel. Cells were then used for functional assays based on RNA and protein extraction.

### Western blot analysis

In brief, whole cell lysates, cytoplasm and nuclear lysates were prepared with a protein extraction kit (Millipore Corporation, USA), and protein concentrations were detected using a Biomate 3s (Thermo Fisher Scientific, USA). First, 5 μg of protein was subjected to electrophoresis on a 10–15% SDS-PAGE gel and then transferred onto polyvinylidene difluoride (PVDF) membrane (Millipore, USA) by electrophoresis. After blocking for 1 h in 5% BSA, the membranes were incubated with antibodies against proteins or β-actin and histone 3 (standard controls) followed by HRP-conjugated secondary antibodies. The signals were detected using the Pierce ECL-plus substrate (Thermo Fisher Scientific, USA) and scanned with a Fluor Chem FC3 camera system (Protein-Simple Co., USA). The images were analysed using Alpha View software (AIC, USA), and quantitative analyses are presented graphically.

### Cell migration and invasion assay

In the wound-healing assay, 2 × 10^5^ cells/well was plated onto 24-well plates. Cells were cultured with 0 or 50 ng/ml of IL-23 for 24 h. After scratching the monolayer with the 10 μl pipette tips (Axygen, USA), cells were washed with PBS, cultured in serum-free DMEM, and photographed after 24 h. For further quantification of the progress of EMT, we used the transwell migration and invasion assay. Before beginning the transwell migration assay, the OC cells were cultured with or without IL-23 at 50 ng/ml for 24 h, after which they were digested and resuspended. Then, 5 × 10^4^ cells were added to each upper chamber in serum-free medium, and migration toward medium containing FBS (the final concentration of 10%) was determined after 24 h. Cells that migrated through the membrane were fixed and stained with Wright Giemsa solution and then counted using light microscopy. In the invasion assay, the lower chambers of matrigel-coated invasion plates were coated with 10 mg/ml fibronectin overnight at 4°C, and cells that invaded through the matrigel was fixed and stained after 48 h and then counted in three randomly selected fields at 200 × magnification.

### Animal experiments

To determine whether the EMT and invasiveness in OC are related with Wnt/catenin in vivo, female nude mice (Comparative Medicine Centre, Yangzhou University, Yangzhou, China) were treated with hypodermic injections into the axillary regions nearly cervical or back (1 × 10^6^ for each injection location) as previously described^38^. The 3 mice injected with physiological saline were taken as negative control group. Mice were maintained under pathogen-free conditions in accordance with the institutional guidelines and approved protocols from the Research Animal Care Committees of Jiangsu University. The mice were observed for tumour formation by palpation. Ten days later, the mice bearing micro-tumours were randomly divided into groups. The control mice in group A (n = 3) were injected with 10% DMSO in 100 μl PBS once per day for 14 days. Each mouse in group B (n = 3) was treated with ICG-001 (50 μmol dissolved with DMSO in 100 μl PBS per day) injected into the caudal vein. In group C (n = 3), IL-23 (50 ng in 100 μl PBS per day) was injected approximately 1 cm from the tumours. Mice in group D (n = 3) were treated with ICG-001 and IL-23 successively at 6 hours daily in the same concentrations used in the other groups. Tumour growth was monitored every 3 days by measuring the length and width of each tumour using callipers. Tumour volumes were calculated using the following formula: length × width × 0.5. All mice were euthanised 30 days later, and tumour tissues were excised. All the above procedures received ethical approval from the Jiangsu University Animal Care and Use Committee. To immunohistochemically analyse the phenotypic variations of the cancer cells in vivo, the sections of subcutaneous xenograft tumours were stained with antibodies against E-cadherin and vimentin. The rest tumours were dissected and subjected to mechanical and enzymatic dissociation for total protein lysates. The expression levels of IL-23R, MMP-9 and VEGF-C were detected by immunoblotting.

### Statistical analysis

The differences between groups in the invasion assay, the real-time PCR, ELISA and animal experiment results, the proteins of transfection with siRNA and the Wnt/β-catenin pathway, the EMT markers, IL-23R and IL-23p19 were analysed using two-way ANOVA. The expression of proliferation in oesophageal cancer cells was analysed by one-way ANOVA. Student's t-test was used to analyse the association of IL-23 expression with OC metastasis status. Difference were considered significant for *p* < 0.05. All data were analysed with GraphPad Prism software (version 5.0).

### Ethical approval

This study was approved by the Ethics Committee of The Hospital Affiliated to Jiangsu University and was performed in accordance with the ethical standards laid down in the 1964 declaration of Helsinki and all subsequent revisions.

## Author Contributions

G.H., S.L., C.X. and T.Z. carried out the PCR and Western blotting. W.L., Y.S. and H.L. carried out the motility assays and immunoassays. Y.Z., Y.S. and W.L. carried out the Elisa and animal experiments. Y.Z. and W.L. participated in the design of the study and performed the statistical analysis. D.C. and C.M. conceived of the study, and wrote the main manuscript text. All authors read and approved the final manuscript.

## Supplementary Material

Supplementary Information Interleukin-23 promotes the epithelial-mesenchymal transition of oesophageal carcinoma cells via the Wnt/β-catenin pathway.

## Figures and Tables

**Table 1:** Correlation of IL-23 expression with clinicopathological features in 23 OC patients

Clinicopathological Features	Number (n = 23)	IL-23^1^(Mean ± SD)	P value
**Gender**			0.6927
Male	15	3.13 ± 2.01	
Female	8	3.83 ± 2.02	
**Age**			0.5850
≤50	4	3.16 ± 1.04	
>50	19	3.53 ± 0.25	
**Tumour size (cm)**			0.4839
≤5	13	3.08 ± 0.88	
>5	10	3.73 ± 1.17	
**Tumour location**			0.7200
Upper/Middle	11	3.53 ± 0.64	
Lower	12	4.11 ± 2.03	
**TNM stage**			0.0517
I–II	18	2.86 ± 0.9	
III–IV	5	5.11 ± 1.12	
**Lymphatic metastasis**^2^			**0.0105**
No	6	2.6 ± 1.04	
Yes	17	6.67 ± 1.16	
**Distant metastasis**^2^			**0.0076**
No	19	3.1 ± 1.02	
Yes	4	8.17 ± 1.44	

^1^Expression levels of IL-23 were detected by immunohistochemical staining.

^2^Difference is considered significant when *p* < 0.05 (shown in bold).
