# Supplementary material for: Interleukin-23 promotes the epithelial-mesenchymal transition of oesophageal carcinoma cells via the Wnt/β-catenin pathway
Source: Sci Rep. 2015 Feb 27;5:8604. doi: 10.1038/srep08604 (PMC4342574; doi:10.1038/srep08604)
Supplement: Supplementary Information — Interleukin-23 promotes the epithelial-mesenchymal transition of oesophageal carcinoma cells via the Wnt/β-catenin pathway. [file srep08604-s1.pdf]

Interleukin-23 promotes the epithelial-mesenchymal transition of oesophageal carcinoma cells via the Wnt/β-catenin pathway

Deyu Chen[1*], Wei Li[1, 3*], Shenzha Liu[1], Yuting Su[1], Guohu Han[1], Chenchen Xu[2], Hongli Liu[2], Tingting Zheng[2], Yuepeng Zhou[1], Chaoming Mao [1, 2]

[1] Institute of Oncology, [2] Department of Nuclear Medicine, the Hospital Affiliated to Jiangsu University, Zhenjiang 212001, China; [3] Department of Oncology, Jintan Hospital Affiliated to Jiangsu University, Changzhou 213200, China.

MATERIALS:

Antibodies: VEGF-C, MMP-9, Snail1, Slug, E-cadherin, Vimentin, β-catenin, Phospho-β-Catenin (Ser33/37/Thr41), Phospho-GSK-3β (Ser9) were purchased from Cell Signal (USA). IL-23R antibody, GSK3-β, β-actin, Histone H3 and HRP-antibody were purchased from Santa Cruz (USA). Angptl2 was purchased from Abcam (USA). IL-23, IL-23p19 antibodies were purchased from R&D Systems (USA).

S1

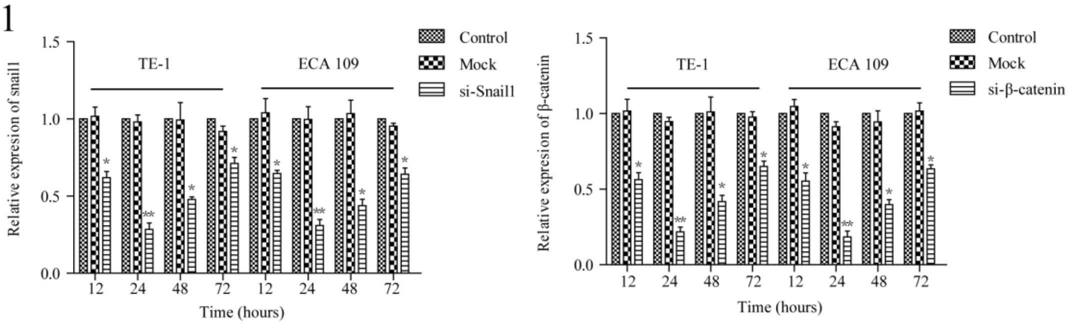

Figure S1. The expression of snail1 and β-catenin by qRT-PCR in groups of cells. The expression of snail1 and β-catenin were analyzed by qRT-PCR with Control, Mock, and transfecting siRNA-snail1 or siRNA-β-catenin at 12h, 24h, 48h, and 72h in TE-1 and ECA109 cells. The resulting data were from at least three independent experiments.

S2

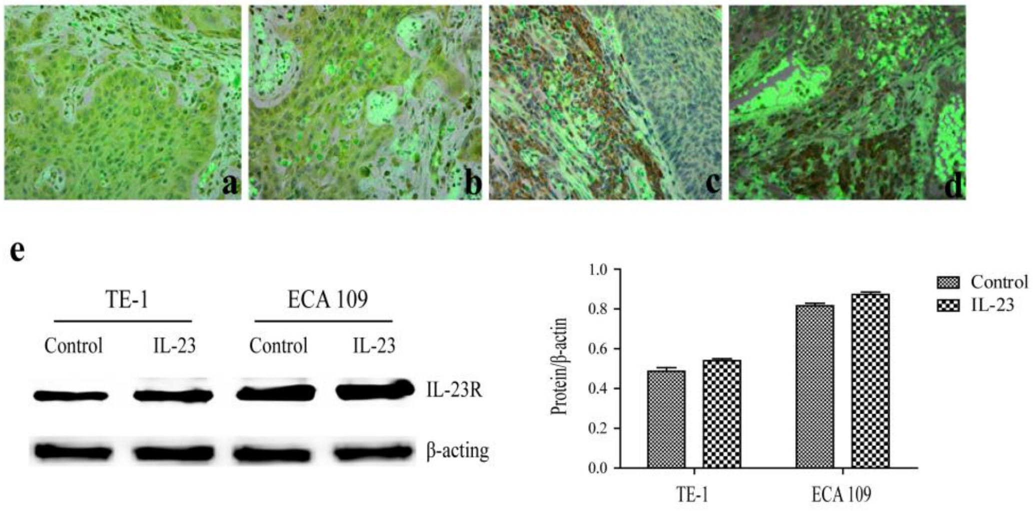

Figure S2. The IL-23R in OC tissues and OSCCs treated with IL-23. The localization of IL-23 was marked in brown by immunohistochemistry and IL-23R in green fluorescence by immunofluorescence (a, b were tumour tissues; c, d were precancerous tissues). In e, the changes of IL-23R in OC cells treated with IL-23 (50 ng/ml) for 24 h were shown. Compared with control, there was no significant difference in the expression of IL-23R in OC cells treated with IL-23. All data derive from at least three independent experiments.

S3

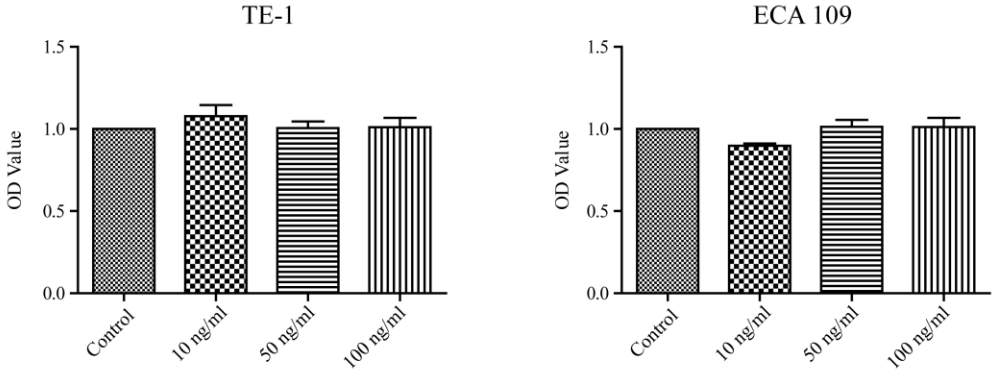

Figure S3. The proliferation in OC cells treated with IL-23. MTT analysis was used to detect the changes of proliferation in OC cells, treated with a different concentration of IL-23 for 24 h. There was no significant difference among them in the proliferation in OC cells treated with IL-23. All data derive from at least three independent experiments.

S4

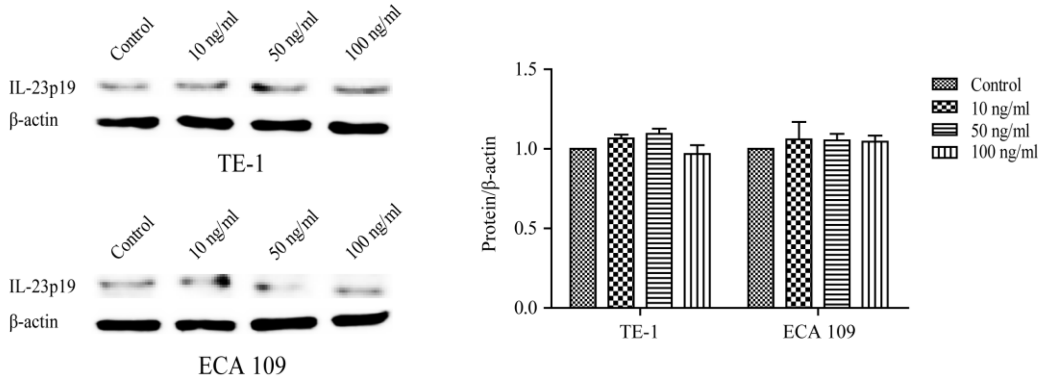

Figure S4. The expression of endogenous IL-23p19 in OC cells treated with IL-23. Treated with a different concentration of IL-23 for 24 h, the expression of endogenous IL-23p19 in OC cells was detected by western blot. As shown in the histograms, there was no significant difference among them. All data derive from at least three independent experiments.
